# LATCH Score for the Identification and Correction of Breastfeeding Problems: A Prospective Observational Study

**DOI:** 10.7759/cureus.101912

**Published:** 2026-01-20

**Authors:** Monika R Boora, Shreedevi Kori, Shobha Shiragur, Ekta Chhabra, Mallanagouda M Patil, Sarvada Umerjikar

**Affiliations:** 1 Obstetrics and Gynaecology, Shri B. M. Patil Medical College, Hospital and Research Centre, BLDE (Deemed to be University), Vijayapura, IND; 2 Paediatrics, Shri B. M. Patil Medical College, Hospital and Research Centre, BLDE (Deemed to be University), Vijayapura, IND

**Keywords:** breastfeeding, breastfeeding effectiveness, lactation counseling, latch score, postnatal care

## Abstract

Background

Breastfeeding is fundamental to infant survival, growth, and development, offering optimal nutrition and immunological protection while conferring long-term health benefits to both infants and mothers. Despite strong evidence supporting exclusive breastfeeding, early breastfeeding difficulties related to improper latch, positioning, and maternal discomfort remain common, particularly in the immediate postnatal period. Early identification and correction of these problems are essential to ensure successful breastfeeding. This study aimed to assess breastfeeding effectiveness using the LATCH (Latch, Audible swallowing, Type of nipple, Comfort, Hold) scoring system in the early postnatal period and to evaluate the impact of LATCH score-guided counseling and hands-on lactation support on breastfeeding outcomes.

Methodology

This prospective observational study was conducted among 229 primiparous mother-infant dyads delivering at term in a tertiary care hospital in Karnataka, India. Breastfeeding effectiveness was assessed using the LATCH scoring system at 1-2 hours postpartum and reassessed at 24-48 hours following structured lactation counseling and hands-on support. Data were analyzed using SPSS version 26.0 (IBM Corp., Armonk, NY, USA), and associations were evaluated using appropriate statistical tests, with p-values <0.05 considered statistically significant.

Results

Most mothers were aged 21-30 years (171; 74.7%). Poor LATCH scores (0-3) were observed in 109 (47.6%) mothers at 1-2 hours postpartum, which reduced to 26 (11.4%) at 24-48 hours. Overall improvement in LATCH scores was noted in 202 (88.2%) mothers. Improvement in LATCH score was significantly associated with antenatal period status (p = 0.021), mode of delivery (p = 0.015), and initial LATCH score at 1-2 hours postpartum (p = 0.016). LATCH assessment at 24-48 hours demonstrated high sensitivity (96.1%) and specificity (99.0%) for predicting improvement in breastfeeding performance.

Conclusions

LATCH score-guided counseling combined with early postnatal hands-on lactation support significantly improved breastfeeding effectiveness within the first 48 hours postpartum. Assessment at 24-48 hours showed high diagnostic accuracy for identifying improvement in breastfeeding performance, supporting the routine use of LATCH scoring in postnatal care.

## Introduction

Breastfeeding is a vital component of infant nutrition and plays a crucial role in promoting optimal growth, development, and survival during early life [1]. Human breast milk provides ideal nutrition along with immunological protection through antibodies, anti-inflammatory agents, and bioactive factors that safeguard infants against infections [2]. In addition to benefiting the infant, breastfeeding enhances maternal-infant bonding, improves maternal confidence, and offers long-term health advantages for mothers [3]. Owing to these benefits, exclusive breastfeeding for the first six months of life is universally recommended [4].

Exclusive breastfeeding has been shown to reduce neonatal and infant morbidity and mortality by lowering the incidence of respiratory and gastrointestinal infections [5]. Components such as secretory immunoglobulin A support mucosal immunity and gastrointestinal maturation, while long-term benefits include improved neurocognitive development and reduced risk of obesity and metabolic disorders [6]. Early initiation of breastfeeding, particularly within the first hour of birth, is associated with a significant reduction in neonatal deaths [7]. Despite strong evidence supporting these benefits, exclusive breastfeeding rates remain suboptimal in many developing countries, including India [8].

Multiple factors contribute to early discontinuation of breastfeeding. Common challenges include improper latch and positioning, ineffective sucking, nipple pain, breast engorgement, maternal anxiety, and perceived insufficient milk supply [9]. Obstetric factors such as cesarean delivery and delayed initiation further complicate breastfeeding practices [10]. Health-system constraints, including early discharge, limited postnatal counseling, and inadequate lactation support, often prevent timely identification and correction of these problems. As a result, preventable breastfeeding difficulties frequently lead to premature cessation of exclusive breastfeeding [11].

The early postnatal period offers a critical opportunity to establish successful breastfeeding practices. Systematic assessment during this time can help identify problems early and guide appropriate interventions. Several breastfeeding assessment tools are available; however, the LATCH scoring system is widely used due to its simplicity and clinical applicability [12]. Introduced in 1994, the LATCH score evaluates five key components, such as Latch, Audible swallowing, Type of nipple, Comfort, and Hold, providing an objective measure of breastfeeding effectiveness [13]. It allows healthcare providers to identify specific problem areas and deliver focused counseling and corrective support [14].

Routine use of the LATCH score is particularly valuable in tertiary care settings, where high-risk mothers and vulnerable neonates such as preterm, low-birth-weight, jaundiced, or hypoglycemic infants require efficient feeding [15]. A low LATCH score alerts caregivers to inadequate milk transfer and enables early intervention, thereby preventing complications and supporting optimal neonatal outcomes. Repeated assessment also helps monitor improvement following counseling and enhances maternal confidence [16].

Although the LATCH score is a validated and practical tool, evidence regarding its routine use in standard postnatal care settings in our region remains limited. Therefore, the present study was undertaken to evaluate the role of the LATCH scoring system in early identification of breastfeeding problems and to assess the effectiveness of LATCH score-guided counseling and interventions on improving breastfeeding outcomes during the early postnatal period.

## Materials and methods

Study design and setting

This prospective observational study was conducted in the Department of Obstetrics and Gynaecology at BLDE (Deemed to be University), Shri B. M. Patil Medical College Hospital and Research Centre, Vijayapura, Karnataka, India. The study commenced in January 2024 and was conducted over a period of 18 months.

Ethical considerations

Institutional Review Board approval was obtained before the initiation of the study (approval number: BLDE (DU)/IEC-SBMPMC/059/202-24). Written informed consent was obtained from all participants before enrolment, and confidentiality of participant data was maintained throughout the study.

Study population

All eligible primiparous women who delivered at term (37-42 completed weeks of gestation) in the labor room during the study period were considered for inclusion.

Inclusion and exclusion criteria

The study included primiparous women who had a term singleton delivery between 37 and 42 completed weeks of gestation and delivered live-born neonates and mothers who were willing to initiate breastfeeding and provided written informed consent. Women were excluded if they had a preterm delivery or a multiple pregnancy, if their neonates required admission to the neonatal intensive care unit, or if there were contraindications to breastfeeding, including maternal HIV positivity or infant galactosemia.

Sample size calculation

The sample size was calculated based on a previous study by Rapheal et al. [16], which reported a prevalence of breastfeeding problems of 11.8% at 24-48 hours postpartum. Using a confidence level of 98%, a level of significance of 2%, and a margin of error of 0.05, the required sample size was calculated using the following standard formula: n = (Z² × p × (1 − p)) / d², where n is the required sample size, Z is the standard normal deviate corresponding to a 98% confidence level (Z = 2.33), p is the expected prevalence of breastfeeding problems (11.8%), and d is the margin of error (0.05). Based on this calculation, a total sample size of 229 participants was included in the study.

Breastfeeding assessment tool

Breastfeeding effectiveness was assessed using the LATCH scoring system. LATCH is an acronym for Latch, Audible swallowing, Type of nipple, Comfort, and Hold [13]. Each component is scored from 0 to 2, yielding a total score ranging from 0 to 10. Lower scores indicate poor breastfeeding effectiveness, while higher scores reflect better latch and feeding technique. Scores of 0-3 were considered poor, 4-7 moderate, and 8-10 good breastfeeding effectiveness.

Study procedure

Breastfeeding assessment using the LATCH scoring system was performed at the following two time points: initially at 1-2 hours postpartum before intervention, and subsequently at 24-48 hours postpartum after intervention.

Following the initial assessment, all mothers received structured breastfeeding education and support. The intervention consisted of individualized counseling, demonstration of correct positioning and attachment, and hands-on training using visual aids and direct observation. Specific breastfeeding problems identified during the initial assessment, such as poor latch, ineffective sucking, or maternal discomfort, were addressed through targeted corrective measures. Mothers who delivered by cesarean section were additionally counseled on breastfeeding in the side-lying position, with emphasis on comfort, positioning, and recognition of effective attachment.

The LATCH score was reassessed at 24-48 hours postpartum following the intervention. Improvement in breastfeeding performance was defined as an increase in the total LATCH score from the initial assessment to reassessment. A LATCH score greater than 8 at 48 hours postpartum was considered suggestive of a high likelihood of continued effective breastfeeding.

Statistical analysis

Data were entered into Microsoft Excel (Microsoft Corp., Redmond, WA, USA) and analyzed using SPSS version 26.0 (IBM Corp., Armonk, NY, USA). Continuous variables were expressed as mean and standard deviation, while categorical variables were presented as frequencies and percentages. Comparisons of continuous variables were performed using the paired sample t-test. Associations between categorical variables were assessed using the chi-square test. A two-tailed p-value <0.05 was considered statistically significant.

Assessment of the sensitivity and specificity of the LATCH score

Sensitivity and specificity analyses were performed to evaluate the ability of the LATCH score assessed at different time points to predict improvement in breastfeeding performance. Improvement in breastfeeding performance was defined as an increase in the total LATCH score between the initial assessment at 1-2 hours postpartum and the reassessment at 24-48 hours postpartum [13]. Mothers showing an increase in LATCH score were classified as “Improved,” while those with no increase were classified as “Not improved.”

Sensitivity and specificity at 1-2 hours postpartum

For the assessment conducted at 1-2 hours postpartum, a low LATCH score (0-3) was considered a positive test for predicting non-improvement in breastfeeding performance. Sensitivity was defined as the ability of a low LATCH score (0-3) at 1-2 hours to correctly identify mothers who did not show improvement in LATCH score at 24-48 hours. Specificity was defined as the ability of a higher LATCH score (4-7) at 1-2 hours to correctly identify mothers who showed improvement in LATCH score at 24-48 hours.

Sensitivity and specificity at 24-48 hours postpartum

For the reassessment at 24-48 hours postpartum, LATCH score categories (0-3, 4-7, and 8-10) were evaluated [16]. Sensitivity was defined as the proportion of mothers who showed improvement and were correctly identified by having a LATCH score ≥4 at 24-48 hours. Specificity was defined as the proportion of mothers who did not show improvement and were correctly identified by having a low LATCH score (0-3) at 24-48 hours. Sensitivity and specificity were calculated using 2×2 contingency tables. No receiver operating characteristic curve analysis was performed.

## Results

The study included 229 primiparous mothers, with most participants belonging to the 21-30-year age group (171, 74.7%). Antenatal breastfeeding counselling was received by 195 (85.2%), and an uneventful antenatal period was observed in 195 (85.2%). Cesarean section was the most common mode of delivery (128, 55.9%), and the majority of newborns had a birth weight ≥2.5 kg (200, 87.3%) (Table 1).

**Table 1:** Baseline maternal and neonatal characteristics of the study population (n = 229). Data are presented as frequency and percentage or mean values. Descriptive statistics were used to summarize maternal age, antenatal characteristics, mode of delivery, and neonatal birth weight.

Variable	Category	n (%)
Maternal age (years)	<20	38 (16.6)
	21–30	171 (74.7)
	31–40	20 (8.7)
Antenatal breastfeeding counseling	Given	195 (85.2)
	Not given	34 (14.8)
Antenatal period status	Uneventful	195 (85.2)
	Eventful	34 (14.8)
Mode of delivery	Cesarean section	128 (55.9)
	Vaginal delivery	101 (44.1)
Birth weight	<2.5 kg	29 (12.7)
	≥2.5 kg	200 (87.3)

Among the 34 mothers with an eventful antenatal period, hypertension was the most frequent complication (12, 35.2%), followed by hypothyroidism (9, 26.4%). Anemia was noted in 5 (14.7%), while blood transfusion and other conditions accounted for 4 (11.7%) each (Table 2).

**Table 2:** Distribution of antenatal complications among eventful pregnancies (n = 34). Data are expressed as frequency and percentage. Descriptive analysis was performed to summarize the pattern of antenatal complications among mothers with eventful antenatal periods. *: Others include cystectomy, myomectomy, and similar conditions.

Antenatal complication	n (%)
Hypertension	12 (35.2)
Hypothyroidism	9 (26.4)
Anemia	5 (14.7)
Blood transfusion	4 (11.7)
Others*	4 (11.7)
Total	34 (100.0)

Early postnatal assessment showed that 109 (47.6%) mothers had poor LATCH scores (0-3) at 1-2 hours, whereas by 24-48 hours, this proportion reduced to 26 (11.4%). Concurrently, good LATCH scores (8-10) were achieved by 91 (39.7%) mothers at 24-48 hours, indicating a clear temporal improvement (Table 3).

**Table 3:** Distribution of LATCH scores at different time points (n = 229). Data are presented as frequency and percentage. LATCH scores assessed at 1–2 hours and 24–48 hours postpartum were descriptively compared to assess temporal changes in breastfeeding effectiveness. LATCH = Latch, Audible swallowing, Type of nipple, Comfort, Hold

LATCH score	1–2 hours, n (%)	24–48 hours, n (%)
0–3	109 (47.6)	26 (11.4)
4–7	120 (52.4)	112 (48.9)
8–10	—	91 (39.7)
Total	229 (100)	229 (100)

Overall improvement in LATCH score following counseling was observed in 202 (88.2%) mothers, while 27 (11.8%) did not show improvement during the study period (Table 4).

**Table 4:** Improvement in LATCH score following counseling (n = 229). Data are expressed as frequency and percentage. Improvement in the LATCH score was assessed by comparing post-counseling scores with the initial assessment. Improvement in LATCH score (increase ≥1 point) was used. LATCH = Latch, Audible swallowing, Type of nipple, Comfort, Hold

LATCH score improvement	n (%)
Yes	202 (88.2)
No	27 (11.8)
Total	229 (100.0)

Component-wise analysis demonstrated an increase in mean scores for all LATCH parameters from day one to day two, with improvements noted across latching, audible swallowing, nipple type, comfort, and positioning/hold components (Table 5).

**Table 5:** Mean (SD) LATCH component scores on day 1 and day 2. Data are presented as mean ± SD. Component-wise LATCH scores were compared using the paired t-test to compare mean scores between day 1 and day 2. SD = standard deviation; LATCH = Latch, Audible swallowing, Type of nipple, Comfort, Hold

LATCH component	Day 1, mean ± SD	Day 2, mean ± SD	Test statistic (t)	P-value
L - Latching	1.47 ± 0.60	1.76 ± 0.43	8.21	<0.001
A - Audible swallowing	1.39 ± 0.55	1.69 ± 0.49	7.94	<0.001
T - Type of nipple	1.44 ± 0.56	1.76 ± 0.43	8.06	<0.001
C - Comfort	1.41 ± 0.52	1.72 ± 0.43	8.34	<0.001
H - Hold	1.36 ± 0.58	1.76 ± 0.43	9.11	<0.001

A statistically significant association with improvement in LATCH score was observed for antenatal period status, mode of delivery, and initial LATCH score at 1-2 hours postpartum. Mothers with uneventful antenatal periods, those who delivered vaginally, and those with lower initial LATCH scores (0-3) demonstrated a higher proportion of improvement following counseling (Table 6).

**Table 6:** Univariate factors associated with improvement in LATCH score. Data are expressed as frequency and percentage. Associations between maternal, obstetric, and LATCH-related variables and improvement in LATCH score were analyzed using the chi-square test. A p-value <0.05 was considered statistically significant. Improvement in LATCH score (increase ≥1 point) was used. LATCH = Latch, Audible swallowing, Type of nipple, Comfort, Hold

Variable	Category	Improved, n (%)	Not improved, n (%)	χ² value	P-value
Antenatal period	Uneventful	176 (90.3)	19 (9.7)	5.33	0.021
	Eventful	26 (76.5)	8 (23.5)		
Mode of delivery	Vaginal	95 (94.1)	6 (5.9)	5.95	0.015
	Cesarean	107 (83.6)	21 (16.4)		
Initial LATCH (1–2 h)	0–3	102 (93.6)	7 (6.4)	5.78	0.016
	4–7	100 (83.3)	20 (16.7)		

Diagnostic accuracy analysis revealed low sensitivity but moderate specificity of the LATCH score at 1-2 hours, whereas assessment at 24-48 hours demonstrated high sensitivity and specificity for predicting improvement in breastfeeding performance (Table 7).

**Table 7:** Diagnostic accuracy of LATCH score for predicting improvement. Sensitivity and specificity were calculated to assess the diagnostic accuracy of LATCH scores at 1–2 hours and 24–48 hours postpartum in predicting improvement in breastfeeding performance. CI = confidence interval; LATCH = Latch, Audible swallowing, Type of nipple, Comfort, Hold

Time of assessment	Sensitivity, % (95% CI)	Specificity, % (95% CI)	Accuracy, %
1–2 hours	6.4 (3.0–11.8)	83.3 (65.3–94.4)	16.2
24–48 hours	96.1 (92.2–98.4)	99.0 (94.6–100)	96.9

## Discussion

The present study evaluated breastfeeding effectiveness using the LATCH scoring system among 229 mother-infant dyads in a tertiary care hospital. Breastfeeding performance was assessed at 1-2 hours and again at 24-48 hours postpartum to determine the impact of structured lactation counseling and hands-on support delivered by trained personnel. The findings provide insight into early breastfeeding dynamics and the factors influencing improvement in LATCH scores during the immediate postnatal period.

The majority of mothers in the present study belonged to the 21-30-year age group, which is considered the optimal reproductive age. This demographic profile is comparable with earlier Indian studies. Halgar et al. reported a mean maternal age of 25 ± 3.82 years [14], while Rapheal et al. observed a mean age of 26.02 years among participating mothers [16]. The similarity in age distribution across studies suggests that breastfeeding challenges and responses to counseling in this age group are representative of the broader reproductive population in tertiary care settings.

A high proportion of mothers in the current study had received antenatal breastfeeding counseling. However, despite this high coverage, the improvement in LATCH scores was comparable between mothers who had received antenatal counseling and those who had not, with no statistically significant difference. This finding is consistent with observations by Halgar et al. and Rapheal et al., who reported that while antenatal counseling improves maternal knowledge and awareness, it is the hands-on postnatal intervention that plays a decisive role in achieving measurable improvement in breastfeeding technique [14,16]. Similarly, Sowjanya et al. demonstrated that LATCH scores improved significantly from the early postnatal period to later follow-up, highlighting the importance of continued support beyond antenatal education [17]. A quasi-experimental study by Lingala et al. also emphasized the benefit of counseling provided at 6-12 hours and reinforced at 24-48 hours postpartum [18].

In the present study, cesarean delivery was the predominant mode of delivery. This distribution is comparable with findings by Halgar et al., who reported nearly equal proportions of vaginal and cesarean deliveries [14]. Mode of delivery showed a statistically significant association with improvement in LATCH scores, with mothers who delivered vaginally demonstrating better improvement compared to those who underwent cesarean section. These findings align with the work of Lamba et al., who reported lower mean LATCH scores in the cesarean delivery group at both the first and 24th hours postpartum compared to the vaginal delivery group [19]. The delayed initiation of breastfeeding, postoperative pain, and restricted mobility following cesarean section may contribute to suboptimal early latch and slower improvement, underscoring the need for targeted lactation support in this subgroup.

Overall, a substantial proportion of mother-infant dyads in the present study demonstrated improvement in LATCH scores following structured counseling and hands-on breastfeeding assistance. This finding is in agreement with Rapheal et al., who observed a significant post-intervention increase in LATCH scores and a marked reduction in the proportion of dyads with scores below 8 between 6-12 hours and 24-48 hours postpartum [16]. Similar results were reported by Halgar et al., where structured support led to a significant increase in mean LATCH scores from 5.83 ± 1.64 to 9.31 ± 1.50, independent of maternal or infant characteristics [14]. These consistent findings across studies reinforce the effectiveness of early postnatal lactation support in improving breastfeeding performance.

An important observation in the current study was that dyads with very low initial LATCH scores (0-3) showed a higher proportion of improvement compared to those with moderate scores. This statistically significant association suggests that early identification of poor latch provides an opportunity for timely and effective intervention. Fadiloglu et al. similarly reported that breastfeeding initiation within 30 minutes of delivery significantly improved LATCH scores at subsequent assessments [20]. Lamba et al. also demonstrated progressive improvement in mean LATCH scores from the first to the 24th hour postpartum in both cesarean and vaginal delivery groups, supporting the benefit of early and repeated assistance [19].

By 24-48 hours postpartum, only a small proportion of mothers in the present study continued to have poor LATCH scores, while a considerable number achieved good scores. This temporal improvement highlights the effectiveness of early breastfeeding counseling, hands-on instruction, and corrective interventions during the immediate postnatal period. Rapheal et al. similarly reported a substantial reduction in the proportion of mothers with LATCH scores below 8 between early and later postpartum assessments in the intervention group [16]. Halgar et al. also demonstrated that low-to-moderate LATCH scores can be transformed into good breastfeeding technique with structured postnatal support [14].

The diagnostic accuracy analysis revealed that LATCH scores assessed at 1-2 hours postpartum had very low sensitivity but good specificity for predicting subsequent improvement. This finding suggests that very early LATCH assessment may not reliably identify mothers who will ultimately experience breastfeeding difficulties, as latch technique and maternal confidence are still evolving immediately after delivery. This observation is consistent with existing literature indicating that early postpartum LATCH measurements have limited predictive value. Sowjanya et al. reported much higher sensitivity and specificity when LATCH scores were assessed at later time points, particularly at 24-48 hours [17]. Shah et al. also noted that LATCH scores at discharge were better predictors of successful breastfeeding outcomes [21].

Limitations of the study

The study was conducted in a single tertiary care hospital, which may limit the generalizability of the findings to rural or community-based settings where access to trained lactation support may be limited. Although the LATCH scoring system is a validated tool, assessments were performed by a limited number of trained observers, which may introduce observer bias.

## Conclusions

The present study demonstrates that structured postnatal lactation counseling combined with hands-on breastfeeding support leads to a significant improvement in breastfeeding effectiveness, as measured by LATCH scores, within the first 48 hours postpartum. Although antenatal breastfeeding counseling improves awareness, measurable improvement in breastfeeding technique was primarily associated with early postnatal interventions, particularly among mothers with lower initial LATCH scores and those who delivered vaginally. Assessment at 24-48 hours postpartum showed higher diagnostic accuracy for identifying improvement in breastfeeding performance compared with very early postpartum evaluation. These findings underscore the importance of implementing systematic, early postnatal lactation support in tertiary care settings to promote effective breastfeeding practices during the immediate postnatal period.
